# Combined Effects of *UGT1A1 and SLCO1B1* Variants on Chinese Adult Mild Unconjugated Hyperbilirubinemia

**DOI:** 10.3389/fgene.2019.01073

**Published:** 2019-10-31

**Authors:** Jie Bai, Lei Luo, Shuang Liu, Chen Liang, Li Bai, Yu Chen, Sujun Zheng, Zhongping Duan

**Affiliations:** ^1^Difficult & Complicated Liver Diseases and Artificial Liver Center, Beijing You An Hospital, Capital Medical University, Beijing, China; ^2^Beijing Municipal Key Laboratory of Liver Failure and Artificial Liver Treatment Research, Beijing, China; ^3^Department of Infectious Diseases, Nanchang University Second Affiliated Hospital, Nanchang, China

**Keywords:** unconjugated hyperbilirubinemia, uridine diphospho-glucuronosyl transferase 1A1, solute carrier organic anion transporter family member 1B, variant, combined effect

## Abstract

The potential for genetic variation to cause adult unconjugated hyperbilirubinemia is increasingly being recognized. However, the cumulative effects of genetic variants have not been fully illuminated. The current study aimed to investigate the effects of uridine diphospho-glucuronosyl transferase 1A1 (*UGT1A1*) and/or solute carrier organic anion transporter family member 1B (*SLCO1B*) polymorphic variants and their combined effects on mild unconjugated hyperbilirubinemia in Chinese adults. Fourteen genetic variants in the *UGT1A1* or *SLCO1B* gene were genotyped through sequencing in 148 adults with unconjugated hyperbilirubinemia and 158 healthy controls. Variants c.-3275T > G, (TA)_6_>(TA)_7_, c.211G > A or c.1091C > T within the *UGT1A1* gene as well as c.521T > C within the *SLCO1B1* gene appear to be genetic risk factors for inherited unconjugated hyperbilirubinemia. After adjusting for covariates, the results of multivariate logistic regressions revealed that odds ratios (ORs) [(with 95% confidence interval (CI)] of these five variants were 2.35 (95% CI: 1.37–4.01, p = 0.002), 2.38 (95% CI: 1.35–4.20, p = 0.003), 2.99 (95% CI: 1.71–5.21, p < 0.001), 7.60 (95% CI: 1.99–28.96, p = 0.003), and 2.54 (95% CI: 1.27–5.11, p = 0.009), respectively. The OR for unconjugated hyperbilirubinemia is positively correlated with the cumulative number of these five variants in adults. And the greater the number of genetic variations, the higher the total bilirubin level. Adults carrying diplotype 3/4 (homozygous c.-3275T > G and heterozygous (TA)_6_>(TA)_7_) had higher bilirubin levels than those with diplotypes 1/3 (heterozygous c.-3275T > G and (TA)_6_>(TA)_7_)) or 1/4 (heterozygous c.-3275T > G) (*P* < 0.05). Similarly, bilirubin levels in individuals with diplotype 2/4 (heterozygous c.-3275T > G and c.211G > A) were higher than adults carrying diplotypes 1/2 (heterozygous c.211G > A) or 1/4 (*P* < 0.001). For subjects with heterozygous or homozygous variant c.211G> A, as the number of c.521T > C alleles variation increased, the incidence of unconjugated hyperbilirubinemia increased, but it was not statistically significant. Our results indicate that variants of *UGT1A1* and/or *SLCO1B1* have combined effects on Chinese adult mild unconjugated hyperbilirubinemia.

## Introduction

Bilirubin is produced mainly by the turnover of red blood cells and then transported to the liver *via* the solute carrier organic anion transporter family member 1B (SLCO1B) (Tiribelli and Ostrow, 1993). In the liver, Uridine diphospho-glucuronosyl transferase 1A1 (UGT1A1) catalyzes the binding of unconjugated bilirubin and glucuronic acid molecules into hydrophilic form that is excreted into bile (Clarke et al., 1997).

Several studies have demonstrated the potential contribution of genetic factors to human serum unconjugated bilirubin levels (Johnson et al., 2009; Dai et al., 2013). Among the genes involved in bilirubin metabolism, *UGT1A1* gene is considered to be a major factor controlling serum bilirubin levels (Johnson et al., 2009). Polymorphisms in the promoter and coding region of the *UGT1A1* gene often underline Gilbert Syndrome (GS), a mild unconjugated hyperbilirubinemia condition (Chiddarwar et al., 2017; Wagner et al., 2018). In Caucasian and African populations, the most common polymorphism of *UGT1A1* is an additional TA repeat in the TATA box region of the promoter, i.e., A(TA)_7_TAA (35.7–41.5%) (Beutler et al., 1998; Huang et al., 2004; Erlinger et al., 2014), while the predominant variation in Asians is a missense mutation, c.211G > A (p.G71R) (11–33.2%) (Chen et al., 2014; Chiddarwar et al., 2017; Yanagi et al., 2017). An exome-wide association study demonstrated that *UGT1A1* intronic variations may also reduce UGT1A1 enzyme activity and cause unconjugated hyperbilirubinemia (Oussalah et al., 2015). Moreover, the compound heterozygous *UGT1A1* variant has also been reported in GS (Skierka et al., 2013), suggesting that *UGT1A1* variant may have a cumulative effect on unconjugated hyperbilirubinemia. However, the evidence for this issue is limited.

Another liver-expressing gene, *SLCO1B*, which is closely related to the transportation of bilirubin and organic anions into hepatocytes, has also been found to play a role in bilirubin homeostasis (D'Silva et al., 2014). Some genome-wide association studies (GWAS) suggested that polymorphisms in *SLCO1B1* and *SLCO1B3* reached genome-wide significance associated with bilirubin levels (Johnson et al., 2009; Sanna et al., 2009; Kang et al., 2010; Dai et al., 2013). Further, *SLCO1B1* variations c.388G > A (p.N130D, rs2306283) and c.521T > C (p.V174A, rs4149056) were also found to be risk factors for neonatal unconjugated hyperbilirubinemia (Huang et al., 2004; D'Silva et al., 2014). But the relationship between these *SLCO1B* polymorphisms and unconjugated hyperbilirubinemia in adults is unclear. Furthermore, the genetic basis, especially the combined effects of *UGT1A1* and *SLCO1B* genes for unconjugated hyperbilirubinemia in Chinese descendants has not been fully illuminated. This study aims to determine the potential effects of liver-expressing *UGT1A1* and *SLCO1B* polymorphic variants and their combined effects on mild non-hemolytic unconjugated hyperbilirubinemia.

## Patients and Methods

### Study Subjects

This is a retrospective case-control study. Adult patients who attended routine health checkups at the second affiliated hospital of Luohe Medical College from June to July in 2017, and with the bilirubin detection results of predominantly unconjugated hyperbilirubinemia (total bilirubin ranged from 17.1 to 85 µmol/l), were enrolled in this study. The 158 control subjects were enrolled from the same health examination people with bilirubin values of less than 17.1 µmol/l. Blood samples were taken in 10–12 h fasting state in the morning and the subjects were kept from strenuous exercise. And clinical records including the gender, age as well as biochemical test results were collected. Exclusion criteria were: (1) a history of chronic liver disease (viral hepatitis, autoimmune liver disease, or cirrhosis and so on); (2) abnormal liver functions (alanine aminotransferase (ALT) or aspartate aminotransferase (AST) > 50 U/L); (3) hemolysis signs (hemoglobin <100 g/L, positive Coomb's test); (4) evidence of infection or biliary obstruction or pregnancy; (5) a history of drug use within 6 months.

This Study Was Approved by the Ethics Committee of Beijing You an Hospital, Capital Medical University and a Written Informed Consent Form Was Obtained From All Participants.

### Molecular Analysis

Genomic DNA was extracted from whole blood using a QIAamp DNA Blood Mini Kit 51106 (QIAGEN, Germany) according to the manufacturer's recommended protocol. The promoter, all 5 exons, exon–intron boundaries, and a region in the distal promoter (the phenobarbital response enhancer module, PBREM) of *UGT1A1* were PCR amplified and sequenced, as reported before (Huang et al., 2004). Similarly, the four known *SLCO1B* polymorphisms (388A > G and 521T > C within the *SLCO1B1* gene, IVS8+2087T > C and g.21074122C > T within the *SLCO1B3* gene) were investigated. The PCR products were purified from agarose gel and sequenced *via* an ABI3730XL sequencer (Applied Biosystems, Foster City, CA, USA).

Selection of SNPs was done based on their association with serum bilirubin levels as well as their prevalence in Asia population (Huang et al., 2005; Kang et al., 2010; Zhang et al., 2012; Dai et al., 2013; Chiddarwar et al., 2017). Finally, a total of 14 SNPs (10 of *UGT1A1* and 4 of *SLCO1B*) were selected. Analysis of linkage disequilibrium (LD) and haplotype were performed by using SNPStats website (https://www.snpstats.net/start.htm). Strong LD was defined as both |D′| and r^2^ > 0.8.

### Statistical Analysis

Continuous variables were expressed as mean and standard deviation (SD) and then statistically evaluated by using Student's t-test. Categorical variables were compared by using the chi-squared (χ^2^) test. Logistic regression models were performed to evaluate the effects of SNPs on hyperbilirubinemia. The odds ratios (ORs) with corresponding 95% confidence interval (CI) were estimated between the case and the control groups by using binary logistic regression models after adjusting for clinical factors (including sex, age, hemoglobin, platelet, and albumin). The relation between log-transformed total bilirubin (TB) and different genotypes was also assessed. All statistical analyses were performed by using SPSS version 23.0 (SPSS, Chicago, IL, USA). A P-value < 0.05 was considered to be statistically significant.

## Results

### Clinical Characteristics

A total of 146 adults with hyperbilirubinemia were enrolled in the case group and 158 adults with bilirubin values of less than 17.1µmol/L in the control group. The clinical features of the case and control groups are shown in **Table 1**. The average total bilirubin of the case group and the control group were 24.93 ± 9.71 µmol/l and 11.83 ± 3.04 µmol/L, respectively. Compared with control subjects, case group had a higher level of hemoglobin (HB) (142.19 ± 14.44 g/L vs 137.24 ± 16.47 g/L, *P* = 0.006) and albumin (ALB) (44.98 ± 3.65 g/L vs 44.12 ± 2.27 g/L, *P* = 0.016), but lower platelet (PLT) values (218.53 ± 54.10×10^9^/L vs 231.38 ± 52.13 × 10^9^/L, *P* = 0.026). Besides, there were no obvious differences between the case and the control groups concerning age, gender, blood routine, and other plasma biochemical results.

**Table 1:** Clinical characteristics of case group and control group.

Parameter	Cases (N = 146)	Controls (N = 158)	*P*
Gender, female (%)	66 (45.21%)	77 (48.73%)	0.538
Age (years)	40.86 ± 12.32	40.00 ± 6.98	0.462
WBC(×10^9^/L)	6.01 ± 1.38	6.33 ± 1.51	0.052
HB (g/L)	142.19 ± 14.44	137.24 ± 16.47	0.006
PLT (×10^9^/L)	218.53 ± 54.10	231.38 ± 52.13	0.026
ALT (U/L)	20.61 ± 10.28	20.06 ± 9.22	0.624
AST (U/L)	19.25 ± 5.05	18.56 ± 4.66	0.217
γ-GT (U/L)	21.87 ± 19.13	20.97 ± 11.88	0.62
ALP (U/L)	69.95 ± 24.82	66.92 ± 17.55	0.218
TB (μmol/L)	24.93 ± 9.71	11.83 ± 3.04	<0.001
DB (μmol/L)	8.95 ± 4.54	4.41 ± 1.20	<0.001
IB (μmol/L)	15.97 ± 6.82	7.46 ± 2.00	<0.001
TP (g/L)	76.07 ± 6.73	75.54 ± 4.02	0.414
ALB (g/L)	44.98 ± 3.65	44.12 ± 2.27	0.016
GLB(g/L)	5.51 ± 3.66	6.10 ± 2.66	0.121
FPG (mmol/L)	4.99 ± 0.80	4.86 ± 0.51	0.106
TC (mmol/L)	4.40 ± 0.84	4.41 ± 0.71	0.896
TG (mmol/L)	1.40 ± 0.78	1.53 ± 1.02	0.211
HDL (mmol/L)	1.32 ± 0.29	1.27 ± 0.28	0.096
LDL (mmol/L)	2.64 ± 0.69	2.65 ± 0.63	0.907
BUN (mmol/L)	4.72 ± 1.03	4.85 ± 1.13	0.319
CR (mmol/L)	67.63 ± 11.58	67.27 ± 12.39	0.803
UA (μmol/L)	324.59 ± 92.30	321.18 ± 87.54	0.824

P-values were calculated between groups by using t-test for continuous variables and the χ^2^ test for categorical variables.

WBC, white blood cell count; HB, hemoglobin; PLT, platelet; ALT, alanine aminotransferase; AST, aspartate aminotransferase; γ-GT, glutamyltransferase; ALP, alkaline phosphatase; TB, total bilirubin; DB, direct bilirubin; IB, indirect bilirubin; TP, total protein; ALB, albumin; GLB, globulin; FPG, fasting plasma glucose; TC, total cholesterol; TG, triglyceride; HDL, high-density lipoprotein; LDL, low-density lipoprotein; BUN, blood urea nitrogen; CR, creatinine; UA, uric acid.

### *UGT1A1* and *SLCO1B* Gene Polymorphisms

The frequencies of *UGT1A1* variations are presented in **Table S1**. The incidence of the two most common variants (either heterozygous or homozygous variants) in the case group was significantly higher than that of the control group [(TA)_6_>(TA)_7_: 41.78% vs 22.78%, P < 0.001; c.211G > A: 50.68% vs 29.11%, P < 0.001]. In addition, 8.22% of cases featured homozygous (TA)_7_ variant, while no controls carried this homozygous variation. The incidence of other six *UGT1A1* variations in the case group was also significantly higher for c.-3275T > G (65.07% vs 43.04%, *P* < 0.001), c.-3152G > A (43.84% vs 23.42%, *P* < 0.001), c.686C > A (6.85% vs 1.90%, *P* = 0.033), IVS1+2842G > T (43.15% vs 23.42%, *P* < 0.001), IVS1+2925T > G (43.15% vs 23.42%, *P* < 0.001), and c.1091C > T (11.49% vs 1.90%, *P* = 0.001), but lower for IVS2+15T > C (5.48% vs 13.29%, *P* = 0.021). The proportion of case subjects with c.1456T > G variant was not statistically different from that of control subjects (3.42% vs 0.63%; *P* = 0.182). Moreover, the three variants c.686C > A, c.1091C > T, and c.1456T > G presented only heterozygous forms or wild-type in cases and controls. The LD pattern across the multiple SNPs of *UGT1A1* is shown in **Table S2**. Strong pairwise LD was observed among the four SNPs within *UGT1A1* [(TA)n repeat, c.-3152G > A, IVS1+2842G > T and IVS1+2925T > G], where each |D′| > 0.8 and r^2^ > 0.8. Moderate LD was present between (TA)n repeat polymorphism and c.-3275T > G (|D′| > 0.8 and r^2=^ 0.4320).

The frequencies of different genotypes of the *SLCO1B* gene observed among the cases and controls are also shown in **Table S1**. The four known polymorphisms (388G > A and 521T > C within *SLCO1B1*, IVS8+2087T > C and g.21074122C > T within *SLCO1B3*) were investigated in this study. However, there was only a statistical difference of the frequency of 521T > C variation between the case and the control groups (24.66% vs 13.92%, *P* = 0.017). A low pairwise LD was found between the four polymorphisms of *SLCO1B* (r^2^ < 0.3) (**Table S3**).

### Effects of *UGT1A1* or *SLCO1B* Gene Variants on Unconjugated Hyperbilirubinemia

Initially, the OR with 95% CI of single suspected variant for hyperbilirubinemia was assessed by using separate binary logistic regression (**Table 2**). Further, the results adjusted for gender, age, hemoglobin, PLT, and albumin were also calculated by multivariate logistic regression models (**Table 2**). Four variants of *UGT1A1* and one variant of *SLCO1B1* revealed statistical significance. Among the variations that significantly increased the risk of hyperbilirubinemia, the first priority in descending order was c.1091C > T (OR = 7.596, 95% CI: 1.992–28.956, *P* = 0.003). There were also strong associations between other common variations in *UGT1A1* and hyperbilirubinemia, including c.-3275T > G (OR = 2.345, 95% CI: 1.371–4.012, *P* = 0.002), (TA)_6_>(TA)_7_ (OR = 2.383, 95% CI: 1.351-4.203, *P* = 0.003), and c.211G > A (OR = 2.985, 95% CI: 1.71–5.211, *P* < 0.001). Variant IVS2+15T > C appeared to reduce the risk of hyperbilirubinemia (OR = 0.278, 95% CI: 0.095–0.82, *P* = 0.02). Specifically, variant c.521T > C within *SLCO1B1* was found to have a significantly increased risk of hyperbilirubinemia (OR = 2.544, 95% CI: 1.267–5.11, *P* = 0.009). The log10-transferred TB of different variants is shown in **Figure S1**. After log10-transferred, the upper limit of the normal TB level is 1.23. In summary, subjects with most homozygous or heterozygous variants of *UGT1A1* or variant c.521T > C of *SLCO1B1* have higher bilirubin values than those with wild type. However, in the cases of variants IVS2+15T > C or IVS7+2087T > C, the opposite is true.

**Table 2:** ORs and 95% CIs for unconjugated hyperbilirubinemia associated with genetic variation.

SNP	OR	95% CI	*P*	OR*	95% CI*	P*
Variant within *UGT1A1* gene						
c.-3275T > G	2.47	1.55–3.92	<0.001	2.35	1.37–4.01	0.002
(TA)_6_>(TA)_7_	2.43	1.48–4.00	<0.001	2.38	1.35–4.20	0.003
c.211G > A	2.50	1.56–4.01	<0.001	2.99	1.71–5.21	<0.001
c.686C > A	3.80	1.02–14.09	0.046	2.19	0.48–10.02	0.312
IVS2 + 15T > C	0.38	0.16–0.88	0.025	0.28	0.09–0.82	0.02
c.1091C > T	6.81	1.95–23.75	0.003	7.60	1.99–28.96	0.003
c.1456T > G	5.57	0.64–48.23	0.119	4.28	0.45–40.59	0.205
Variant within *SLCO1B* gene						
*c*.388G > A	1.25	0.51–3.06	0.62	1.11	0.40–3.10	0.84
c.521T > C	2.02	1.13–3.64	0.02	2.54	1.27–5.11	0.009
IVS7 + 2087T > C	0.74	0.19–2.80	0.65	1.20	0.22–6.56	0.834
g.21074122C > T	0.68	0.38–1.204	0.186	0.72	0.36–1.42	0.34

*Adjusted with gender, age, HB, PLT, ALB, WBC, FPG, and HDL. SNP, single nucleotide polymorphism; OR, odds ratio; CI, confidence interval.

**Figure 1:**
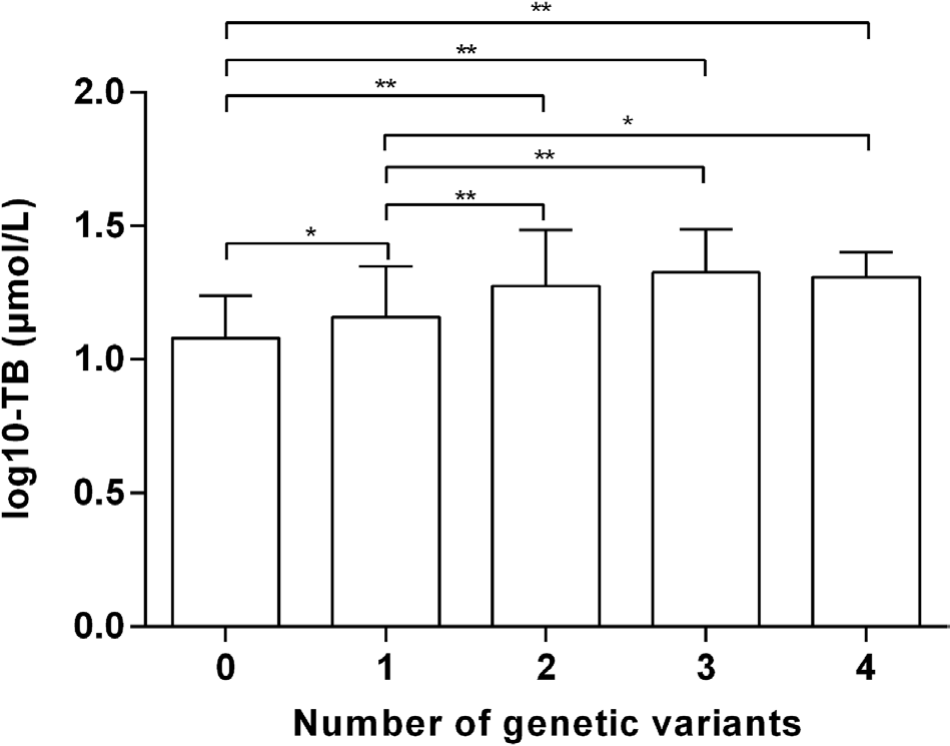
Total bilirubin values (log10-transformed) amongst different numbers of genetic variants. TB, total bilirubin. *P < 0.05 **P < 0.01.

To assess the impact of co-expression of *UGT1A1* or *SLCO1B* variants, the relationship between the number of genetic variants and the risk of unconjugated hyperbilirubinemia was investigated. The OR is positively correlated with the cumulative number of five variants in individuals (**Table 3**). Adjust ORs in adults with one, two, three, and four risk variants were 3.98 (95% CI: 1.48–10.69, *P* = 0.006), 10.92 (95% CI: 3.91–30.53, *P* < 0.001), 18.43 (95% CI: 5.95–57.04, *P* < 0.001) and 34.38 (95% CI: 3.05–387.32, *P* = 0.004), respectively. Besides, the greater the number of genetic variations, the higher the total bilirubin level (**Figure 1**). These data indicated that different variants in *UGT1A1* or *SLCO1B* have combined effects on unconjugated hyperbilirubinemia.

**Table 3:** Adjusted ORs and 95% CI of unconjugated hyperbilirubinemia associated with the number of genetic variation.

Number of genetic variants^a^	Cases (N = 146)	Controls (N = 158)	OR	95% CI	P
0	10	45	1.00	–	–
1	38	68	3.98	1.48–10.69	0.006
2	54	30	10.92	3.91–30.53	<0.001
3	39	13	18.43	5.95–57.04	<0.001
4	5	2	34.38	3.05–387.32	0.004

All results were adjusted with gender, age, HB, PLT, ALB, WBC, FPG, and HDL.

a Variants including c.-3275T > G, (TA)_6_>(TA)_7_, c.211G > A, and c.1091C > T in UGT1A1 as well as variant c.521T > C in SLCO1B1.

OR, odds ratio; CI, confidence interval.

### Combined Effects of *UGT1A1* Variants on Unconjugated Hyperbilirubinemia

To further analyze the combined effects of *UGT1A1* variations on unconjugated hyperbilirubinemia, haplotype and diplotype analyses were performed. Based on multivariate logistic regression results, four SNPs of *UGT1A1*, which increased the risk of hyperbilirubinemia, formed 5 common haplotypes with frequencies > 1% (**Table 4**). Among them, haplotype 1 [–3275T–(TA)_6_–211G–1091C] is the most common (43.52%), representing wild type. Compared with haplotype 1, haplotypes 2 [–3275T–(TA)_6_–211A–1091C], 3 [–3275G–(TA)_7_–211G–1091C], 4 [–3275G–(TA)_6_–211G–1091C] and 5 [–3275G–(TA)_6_–211G–1091A] all increased the risk of unconjugated hyperbilirubinemia (P < 0.05).

**Table 4:** ORs and 95% CIs for unconjugated hyperbilirubinemia associated with *UGT1A1* haplotypes.

Haplotype	Variant							OR (95%CI)	*P*	OR (95%CI)*	P*
	c.-3275T > G	(TA)_6_> (TA)_7_	c.211G > A	c.1091C > T	Cases, frequency	Controls, frequency	Total, frequency				
1	T	(TA)_6_	G	C	26.15%	59.49%	43.52%	1.00	–	1.00	–
2	T	(TA)_6_	A	C	30.70%	15.82%	22.93%	4.51 (2.77–7.35)	<0.001	5.72 (3.19–10.26)	<0.001
3	G	(TA)_7_	G	C	25.00%	11.39%	17.93%	5.29 (3.07–9.11)	<0.001	6.24 (3.29–11.86)	<0.001
4	G	(TA)_6_	G	C	11.86%	12.34%	12.07%	2.07 (1.16–3.68)	0.014	2.27 (1.15–4.47)	0.019
5	G	(TA)_6_	G	T	5.82%	0.95%	3.29%	12.48 (3.36–46.38)	<0.001	16.64 (3.89–71.12)	<0.001

*Adjusted with gender, age, HB, PLT, ALB, WBC, FPG, and HDL. OR, odds ratio; CI, confidence interval.

**Figure 2:**
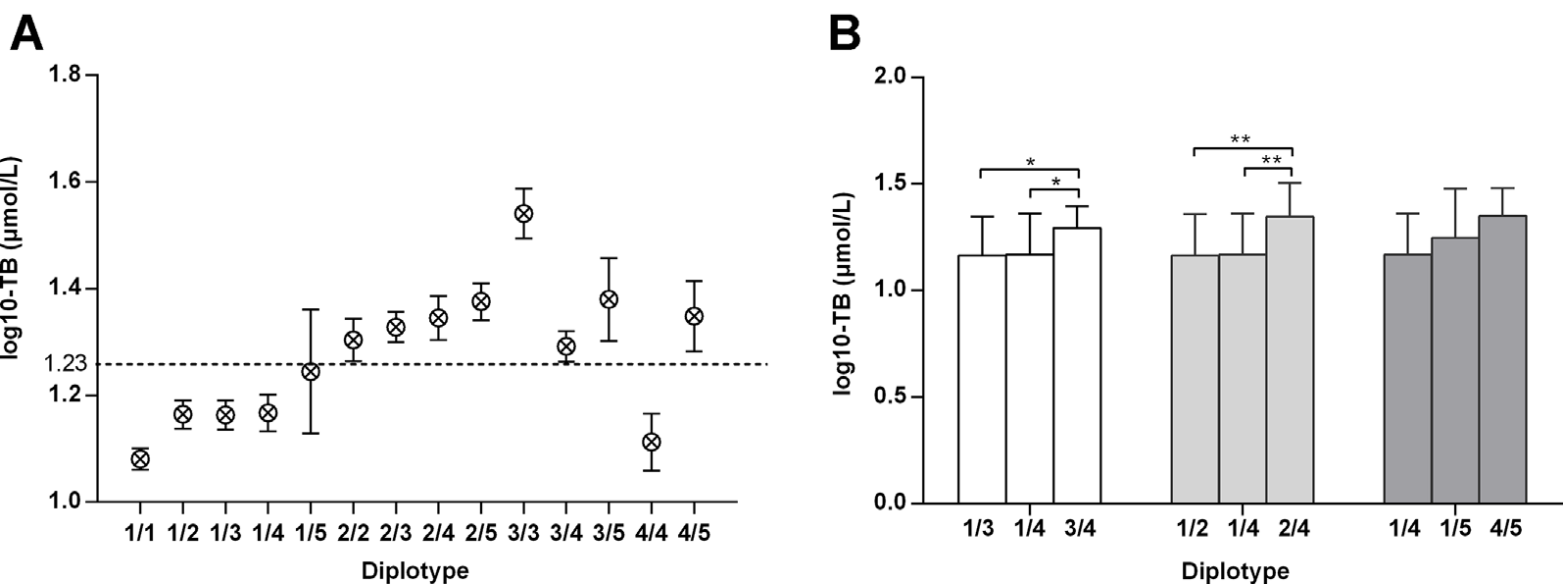
Total bilirubin values (log10-transformed) amongst adults with various *UGT1A1* genotypes. a: –3275T–(TA)_6_–211G–1091C; b: –3275T–(TA)_6_–211A–1091C; c: –3275G–(TA)_7_–211G–1091C; d: –3275G–(TA)_6_–211G–1091C; e: –3275G–(TA)_6_–211G–1091A. *: *P* < 0.05 **: *P* < 0.01.

All subjects were divided into 14 diplotypes, covering a total of 303 (99.7%) adults. All bilirubin values (log10-transformed) among *UGT1A1* diplotypes are shown in **Figure 2A**. Except for 4/4, subjects with homozygous variants or compound heterozygous variants (2/2, 2/3, 2/4, 2/5, 3/3, 3/4, 3/5, 4/5) had the highest levels of total bilirubin, followed by those with heterozygous variants (1/2, 1/3, 1/4, 1/5), while wild type (1/1) the lowest. Of all *UGT1A1* diplotypes, 3/3 (homozygous variation of c.-3275T > G and (TA)_6_>(TA)_7_) featured the highest levels of bilirubin. The diplotypes 1/4 and 4/4 represent heterozygous or homozygous c.-3275T > G variant, respectively, and their bilirubin levels are lower than normal. Moreover, adults carrying diplotype 3/4 (homozygous c.-3275T > G and heterozygous (TA)_6_>(TA)_7_) had higher bilirubin levels than those with diplotypes 1/3 (heterozygous c.-3275T > G and (TA)_6_>(TA)_7_)) or 1/4 (heterozygous c.-3275T > G) (*P* < 0.05) (**Figure 2B**). Similarly, bilirubin levels in individuals with diplotype 2/4 (heterozygous c.-3275T > G and c.211G > A) were higher than those of adults carrying diplotypes 1/2 (heterozygous c.211G > A) or 1/4 (*P* < 0.001). However, there was no significant difference in bilirubin levels among adults with diplotype 1/4, 1/5 or 4/5. These data demonstrated an additive effect of variants c.-3275T > G and (TA)_6_>(TA)_7_ or c.211G > A on unconjugated hyperbilirubinemia.

### Combined Effects of *UGT1A1* and *SLCO1B* Variants on Unconjugated Hyperbilirubinemia

The association between the two common variations (TA)_6_/(TA)_7_ and c.211G > A in *UGT1A1* and variation c.521T > C in *SLCO1B* was further explored. In *UGT1A1*-normal subjects, homozygous variation c.521T > C increased total bilirubin level and the incidence of unconjugated hyperbilirubinemia compared to wild-type or heterozygous forms (**Table S4**). When carried heterozygous variation c.211G > A, the total bilirubin level of heterozygous c.521T > C adults was higher than that of wild type (22.92µmol/L vs 17.21µmol/L, *P* = 0.046); as the number of variation c.521T > C alleles increased, the incidence of unconjugated hyperbilirubinemia increased, but it was not statistically significant (*P* = 0.186). When carried homozygous variation c.211G > A, the incidence of hyperbilirubinemia for individuals featured c.521T > C was higher, but it did not reach statistically significant (100% vs 75%, *P* = 0.214). In addition, among the 21 adults with the homozygous c.211G > A, all 5 subjected with the heterozygous variation c.521T > C suffered from hyperbilirubinemia. But for (TA)_6_>(TA)_7_, we did not observe similar results. These data indicated a cumulative effect of variants c.521T > C and c.211G > A on unconjugated hyperbilirubinemia.

## Discussion

GS, a benign condition that occurs in 5–10% of the population (Wagner et al., 2018), is characterized by mild unconjugated hyperbilirubinemia, in the absence of liver disease or hemolysis (Strassburg, 2010). Although it has long been considered a benign condition and does not require treatment, recent data suggest that affected individuals may be prone to liver damage following various drugs and xenobiotic treatments (Ehmer et al., 2012). Molecular defects in genes involved in the bilirubin metabolic pathway account for hyperbilirubinemia. In the present study, we evaluated the effects of *UGT1A1* and *SLCO1B* on mild unconjugated hyperbilirubinemia in adults. Our results indicated that variations of *UGT1A1* and *SLCO1B* genes had a substantial impact on unconjugated bilirubin levels in Chinese adults. Notably, variant c.521T > C in the *SLCO1B* may be independent genetic risk factors for inherited unconjugated hyperbilirubinemia. Furthermore, the risk of unconjugated hyperbilirubinemia is positively correlated with the cumulative number of variants in adults. To our knowledge, this is the first report that showed the combined effect of *UGT1A1* and *SLCO1B* variants on mild unconjugated hyperbilirubinemia in adults.

Two common variants (TA)_6_>(TA)_7_ and c.211G > A of *UGT1A1* both reduce UGT1A1 enzyme activity and increase unconjugated bilirubin level (Sato et al., 1996; Beutler et al., 1998; Maruo et al., 2016). Our results confirmed the strong association of variants (TA)_6_>(TA)_7_ and c.211G > A with the incidence of unconjugated hyperbilirubinemia. Additionally, the frequencies of variants (TA)_6_>(TA)_7_ and c.211G > A in the case group were significantly higher than those in the control group. And subjects with these two heterozygous or homozygous variants had higher mean total serum bilirubin levels than adults carrying the wild type.

Other mutations occurring in the coding region of *UGT1A1* c.686C > A, c.1091C > T and c.1456T > G can also lead to Gilbert syndrome amongst the Asian population (Huang et al., 2005; Yang et al., 2016). In this study, the frequencies of variants c.686C > A and c.1091C > T in the *UGT1A1* gene in cases and controls were statistically different, but only the OR of c.1091C > T was statistically significant when adjusting the covariates. The role of variants c.686C > A and c.1456T > G needs to be explored through large-scale investigations. Besides, we found that variant IVS2+15T > C appears to reduce the risk of hyperbilirubinemia, and is associated with lower bilirubin levels. However, there is no relevant evidence and further research is needed.

Earlier studies suggested the decrease in transcription caused by both (TA)_6_>(TA)_7_ and c.-3275T > G mutations together may be essential to the Gilbert syndrome (Maruo et al., 2004). In the present study, variation c.-3275T > G was found to be moderately linked with TA repeats. The results of multivariate logistic regression and haplotype analysis demonstrated that variant c.-3275T > G was an independent risk factor of unconjugated hyperbilirubinemia. Interestingly, subjects carrying homozygous or heterozygous variant c.-3275T > G (4/4 or 1/4 diplotype) featured normal mean bilirubin levels. This result is consistent with other researches, suggesting that variant c.-3275T > G was not associated with increased bilirubin levels (Chen et al., 2014; Pasternak et al., 2017). In addition, diplotype analyses revealed that subjects with co-inheriting heterozygous variants c.-3275T > G as well as (TA)_6_>(TA)_7_ or c.211G > A had higher bilirubin levels than those carrying any of the above heterozygous variants. These data indicate a combined effect on unconjugated hyperbilirubinemia for different variants in *UGT1A1*.

Some GS cases have been reported in which plasma clearance of cholephilic dyes such as sulfobromophthalein (BSP) and indocyanine green (ICG) was markedly impaired, indicating a role of impaired hepatic bilirubin uptake (Metreau et al., 1977; Gentile et al., 1990). Genetic analyses suggest that such cases of unconjugated hyperbilirubinemia could be linked to polymorphisms in *SLCO1B1* and *SCLO1B3* (Sanna et al., 2009; Watchko, 2013; Erlinger et al., 2014). In the present research, four polymorphisms of *SLCO1B* identified by previous GWAS were detected (Johnson et al., 2009; Sanna et al., 2009; Kang et al., 2010; Dai et al., 2013). A novel founding is that variant c.521T > C in *SLCO1B1* is closely related to bilirubin levels and significantly increases the risk of unconjugated hyperbilirubinemia (OR = 2.54, 95% CI: 1.27–5.11, *P* = 0.009). Besides, the variant c.521T > C showed an additive effect on *UGT1A1* gene variants, particularly the variant c.211G > A. These results indicated that the change of amino acid (valine to alanine, encoded by nucleotides 520–522) at codon 174 of *SLCO1B1* may reduce the function in unconjugated bilirubin transportation. However, the function of the variants should be validated in cell and animal model experiments. Interestingly, simultaneous homozygous mutations in *SLCO1B1* and *SLCO1B3*, resulting in disruption of hepatic reuptake of bilirubin, may account for the predominantly conjugated hyperbilirubinemia of Rotor syndrome (van de Steeg et al., 2012). These findings suggested that the function of solute carrier organic anion transporter is more complex than transporting unconjugated bilirubin, and thus mutations in these coding genes result in different hyperbilirubinemia propensities.

Besides, previous researches have shown that genes expressed outside the liver also affect bilirubin homeostasis, including glucose-6-phosphate dehydrogenase (G6PD), heme oxygenase (HMOX), biliverdin reductase A (BLVRA), nucleoporin 153 (NUP153) and *UGT1A6* (Huang et al., 2002; Huang et al., 2005; Datta et al., 2012; Oussalah et al., 2015; Chiddarwar et al., 2017). However, our study mainly discussed the effects of intrahepatic genetic variations on non-hemolytic unconjugated hyperbilirubinemia. More studies are encouraged to further explore the effect of genetic variation of extrahepatic expressed genes on Chinese adult unconjugated hyperbilirubinemia.

This study has some limitation. First, the level of bilirubin in GS is fluctuating, especially in particular conditions such as prolonged fasting, fever, and strenuous exercise (Vitek et al., 2019). Although we standardized blood collection and adjusted clinical factors as covariables for all association tests, there are still uncontrollable factors affecting bilirubin levels, such as mood and season. Second, the study is a descriptive study. Based on previous GWAS, our study investigated the *UGT1A1* and *SLCO1B* genetic variation of Chinese adults with hyperbilirubinemia and emphatically explored the combined effects of multiple variants. Consistent with the GWAS, our study confirmed the role of the single genetic variation. Moreover, we found that the variants of *UGT1A1* and *SLCO1B1* have combined effects on the level of bilirubin. The function of the variants should be validated in cell and animal model experiments.

In conclusion, this study demonstrates the impact of genetic factors on non- hemolytic unconjugated hyperbilirubinemia in Chinese population. Variations c.-3275T > G, (TA)_6_>(TA)_7_, c.211G > A, and c.1091C > T within *UGT1A1* as well as c.521T > C within *SLCO1B1* are independent risk factors of mild unconjugated hyperbilirubinemia. Our results also reveal that there is a combined effect on unconjugated hyperbilirubinemia for c.-3275T > G as well as (TA)_6_>(TA)_7_ or c.211G > A in *UGT1A1*. Further, co-expression of c.211G > A of *UGT1A1* and c.521T > C of *SLCO1B1* has a cumulative effect on unconjugated hyperbilirubinemia.

## Data Availability Statement

The raw data supporting the conclusions of this manuscript will be made available by the authors, without undue reservation, to any qualified researcher.

## Ethics Statement

The studies involving human participants were reviewed and approved by Ethics committee of Beijing Youan Hospital, Capital Medical University. The patients/participants provided their written informed consent to participate in this study. Written informed consent was obtained from the individual(s) for the publication of any potentially identifiable images or data included in this article.

## Author Contributions

JB, LL, SZ, and ZD: Conception and design. LL: Patient recruitment. JB and CL: Collection of data. SL and LB: Analysing data. JB: Manuscript draft. JB, LL, YC, SZ and ZD: Editing and revision. All authors read and approved the final manuscript.

## Funding

This study was supported by Beijing Municipal Science and Technology Project (No.Z171100002217070), National Key R&D Program of China (No.2017YFA0103000), National Science and Technology Key Project on “Major Infectious Diseases such as HIV/AIDS, Viral Hepatitis Prevention and Treatment” (NO. 2012ZX10002004-006, No.2017ZX10203201-005, 2017ZX10201201, No.2017ZX10202203-006-001 and No.2017ZX10302201-004-002), “Beijing Municipal Administration of Hospitals” Ascent Plan (No. DFL20151601), Beijing Municipal Administration of Hospitals Clinical Medicine Development of Special Funding Support (No.ZYLX201806), the Digestive Medical Coordinated Development Center of Beijing Municipal Administration of Hospitals (No.XXZ0503), the You An fund for liver diseases and AIDS (YNKTTS201801189), and the Basic-Clinical Cooperation Project of Capital Medical University (17JL47). Science and technology innovation service capacity building - high-precision discipline construction project (11920703).

## Conflict of Interest

The authors declare that the research was conducted in the absence of any commercial or financial relationships that could be construed as a potential conflict of interest.
