# Supplementary material for: Combined Effects of UGT1A1 and SLCO1B1 Variants on Chinese Adult Mild Unconjugated Hyperbilirubinemia
Source: Front Genet. 2019 Oct 31;10:1073. doi: 10.3389/fgene.2019.01073 (PMC6834774; doi:10.3389/fgene.2019.01073)
Supplement: Supplementary file 1 [file DataSheet_1.docx]

**Table S1. The comparison of *UGT1A1* and *SLCO1B*** **gene variations between case and control groups**

| SNP | Variants | location | Genotypes | Cases (N = 146) | Controls (N = 158) | *P* (chi-square test) |
| --- | --- | --- | --- | --- | --- | --- |
| rs4124874 | c.-3275T>G | *UGT1A1*-PBREM | TT | 51 (34.93%) | 90 (56.96%) |  |
|  |  |  | Any variation | 95 (65. 07%) | 68 (43.04%) | <0.001 |
|  |  |  | TG | 64 (43.84%) | 58 (36.71%) |  |
|  |  |  | GG | 31 (21.23%) | 10 (6.33%) |  |
| rs10929302 | c.-3152G>A | *UGT1A1*-PBREM | GG | 82 (56.16%) | 121 (76.58%) |  |
|  |  |  | Any variation | 64 (43.84%) | 37 (23.42%) | <0.001 |
|  |  |  | GA | 52 (35.62%) | 37 (23.42%) |  |
|  |  |  | AA | 12 (8.22%) | 0 |  |
| rs8175347 | (TA)_6_>(TA)_7_ | *UGT1A1*-promoter | TA_6_/TA_6_ | 85 (58.22%) | 122 (77.22%) |  |
|  |  |  | Any variation | 61 (41.78%) | 36 (22.78%) | <0.001 |
|  |  |  | TA_6_/TA_7_ | 49 (33.56%) | 36 (22.78%) |  |
|  |  |  | TA_7_/TA_7_ | 12 (8.22%) | 0 |  |
| rs4148323 | c.211G>A | *UGT1A1-*Exon 1 | GG | 72 (49.32%) | 112 (70.89%) |  |
|  |  |  | Any variation | 74 (50.68%) | 46 (29.11%) | <0.001 |
|  |  |  | GA | 57 (39.04%) | 42 (26.58%) |  |
|  |  |  | AA | 17 (11.64%) | 4 (2.53%) |  |
| rs35350960 | c.686C>A | *UGT1A1-*Exon 1 | CC | 136 (93.15%) | 155 (98.10%) |  |
|  |  |  | Any variation | 10 (6.85%) | 3 (1.90%) | 0.033 |
|  |  |  | CA | 10 (6.85%) | 3(1.90%) |  |
| rs6742078 | IVS1 + 2842G>T | *UGT1A1-*Intron 1 | GG | 83 (56.85%) | 121 (76.58%) |  |
|  |  |  | Any variation | 63 (43.15%) | 37 (23.42%) | <0.001 |
|  |  |  | GT | 51 (34.93%) | 37 (23.42%) |  |
|  |  |  | TT | 12 (8.22%) | 0 |  |
| rs4148324 | IVS1 + 2925T>G | *UGT1A1-*Intron 1 | TT | 83 (56.85%) | 121 (76.58%) |  |
|  |  |  | Any variation | 63 (43.15%) | 37 (23.42%) | <0.001 |
|  |  |  | TG | 51 (34.93%) | 37 (23.42%) |  |
|  |  |  | GG | 12 (8.22%) | 0 |  |
| rs4148327 | IVS2 + 15T>C | *UGT1A1-*Intron 2 | TT | 138 (94.52%) | 137 (86.71%) |  |
|  |  |  | Any variation | 8 (5.48%) | 21 (13.29%) | 0.021 |
|  |  |  | TC | 8 (5.48%) | 20 (12.66%) |  |
|  |  |  | CC | 0 | 1 (0.63%) |  |
| rs34946978 | c.1091C>T | *UGT1A1-*Exon 4 | CC | 129 (88.36%) | 155 (98.10%) |  |
|  |  |  | Any variation | 17 (11.64%) | 3 (1.90%) | 0.001 |
|  |  |  | CT | 17 (11.64%) | 3 (1.90%) |  |
| rs34993780 | c.1456T>G | *UGT1A1-*Exon 5 | TT | 141 (96.58%) | 157 (99.37%) |  |
|  |  |  | Any variation | 5 (3.42%) | 1 (0.63%) | 0.182 |
|  |  |  | TG | 5 (3.42%) | 1 (0.63%) |  |
| rs2306283 | c*.*388G>A | *SLCO1B1-*Exon 5 | GG | 9 (6.16%) | 12 (7.59%) |  |
|  |  |  | Any variation | 137 (93.84%) | 146 (92.41%) | 0.623 |
|  |  |  | GA | 70 (47.95%) | 56 (35.44%) |  |
|  |  |  | AA | 67 (45.89%) | 90 (56.96%) |  |
| rs4149056 | c.521T>C | *SLCO1B1-*Exon 6 | TT | 110 (75.34%) | 136 (86.08%) |  |
|  |  |  | Any variation | 36 (24.66%) | 22 (13.92%) | 0.017 |
|  |  |  | TC | 31 (21.23%) | 21 (13.29%) |  |
|  |  |  | CC | 5 (3.42%) | 1 (0.63%) |  |
| rs2417940 | IVS7 + 2087T>C | *SLCO1B3-*Intron 7 | TT | 5 (3.42%) | 4 (2.53%) |  |
|  |  |  | Any variation | 141 (96.58%) | 153 (96.84%) | 0.912 |
|  |  |  | TC | 49 (33.56%) | 37 (23.42%) |  |
|  |  |  | CC | 92 (63.01%) | 116 (73.42%) |  |
| rs2117032 | g.21074122C>T | *SLCO1B3-*3'-UTR | CC | 33 (22.60%) | 26 (16.46%) |  |
|  |  |  | Any variation | 113 (77.40%) | 131 (82.91%) | 0.184 |
|  |  |  | CT | 67 (45.89%) | 77 (48.73%) |  |
|  |  |  | TT | 46 (31.51%) | 54 (34.18%) |  |

Abbreviations: SNP, single nucleotide polymorphism; PBREM, phenobarbital response enhancer module; UTR, untranslated region.

**Table S2. Linkage disequilibrium (LD) between different SNPs of *UGT1A1* gene**

| \|D’\| | c.-3152G>A | (TA)6>(TA)7 | c.211G>A | c.686C>A | IVS1+2842G>T | IVS1+2925T>G | IVS2+15T>C | c.1091C>T | c.1456T>G |
| --- | --- | --- | --- | --- | --- | --- | --- | --- | --- |
| c.-3275T>G | 0.9996 | 0.9993 | 0.9649 | 0.9953 | 0.9997 | 0.9997 | 0.9967 | 0.9972 | 0.0816 |
| c.-3152G>A | - | 0.9887 | 0.9984 | 0.9962 | 0.9997 | 0.9997 | 0.9941 | 0.1604 | 0.1220 |
| (TA)6>(TA)7 | - | - | 0.9980 | 0.9962 | 0.9887 | 0.9887 | 0.9939 | 0.4421 | 0.1337 |
| c.211G>A | - | - | - | 0.9866 | 0.9984 | 0.9984 | 0.9953 | 0.9943 | 0.0221 |
| c.686C>A | - | - | - | - | 0.9962 | 0.9962 | 0.9371 | 0.9056 | 0.7650 |
| IVS1+2842G>T | - | - | - | - | - | 0.9997 | 0.9941 | 0.5098 | 0.1250 |
| IVS1+2925T>G | - | - | - | - | - | - | 0.9941 | 0.5098 | 0.1250 |
| IVS2+15T>C | - | - | - | - | - | - | - | 0.9621 | 0.8982 |
| c.1091C>T | - | - | - | - | - | - | - | - | 0.0963 |
| r^2^ |  |  |  |  |  |  |  |  |  |
| c.-3275T>G | 0.4517 | 0.4320 | 0.1419 | 0.0428 | 0.4469 | 0.4469 | 0.0260 | 0.0700 | 0.0002 |
| c.-3152G>A | - | 0.9353 | 0.0687 | 0.0950 | 0.9886 | 0.9886 | 0.0117 | 0.0002 | 0.0009 |
| (TA)6>(TA)7 | - | - | 0.0657 | 0.0993 | 0.9458 | 0.9458 | 0.0112 | 0.0015 | 0.0011 |
| c.211G>A | - | - | - | 0.0064 | 0.0680 | 0.0680 | 0.0155 | 0.0102 | 0.0000 |
| c.686C>A | - | - | - | - | 0.0960 | 0.0960 | 0.0010 | 0.0006 | 0.0002 |
| IVS1+2842G>T | - | - | - | - | - | 0.9994 | 0.0116 | 0.0020 | 0.0009 |
| IVS1+2925T>G | - | - | - | - | - | - | 0.0116 | 0.0020 | 0.0009 |
| IVS2+15T>C | - | - | - | - | - | - | - | 0.0016 | 0.0006 |
| c.1091C>T | - | - | - | - | - | - | - | - | 0.0036 |

| \|D’\| | c. 521T>C | IVS8+2087T>C | g.21074122C>T |
| --- | --- | --- | --- |
| c.388A>G | 0.0629 | 0.1778 | 0.1818 |
| c. 521T>C |  | 0.0531 | 0.0248 |
| IVS8+2087T>C |  |  | 0.8668 |
| r^2^ |  |  |  |
| c.388A>G | 0.0012 | 0.0025 | 0.0094 |
| c. 521T>C |  | 0 | 0 |
| IVS8+2087T>C |  |  | 0.2045 |

**Table S3. Linkage disequilibrium (LD) between different SNPs of *SLCO1B* gene**

**Table S4. Clinical Data for the adults with different *UGT1A1* and *SLCO1B1* genotypes**

|  |  |  | Total | *SLCO1B1* | | | *P* | *P* ^Wild-type vs T/C^ | *P*^Wild-type vs C/C^ | *P* ^T/C vs C/C^ |
| --- | --- | --- | --- | --- | --- | --- | --- | --- | --- | --- |
|  |  |  |  | Wild-type | 521T/C | 521C/C |  |  |  |  |
| TB (μmol/L) | *UGT1A1* | Wild-type | 14.56 ± 8.05 | 14.02 ± 5.32 | 12.61 ± 4.12 | 38.33 ± 34.53 | <0.00 ^a^ | 0.418^b^ | <0.001^b^ | 0.326^b^ |
|  |  | (TA)_6_/(TA)_7_ | 17.66 ± 7.12 | 17.67 ± 7.68 | 17.92 ± 5.37 | 13.30 | 0.825^a^ | - | - | - |
|  |  | (TA)_7_/(TA)_7_ | 36.98 ± 13.40 | 40.24 ± 13.65 | 27.20 ± 7.20 | - | 0.152^b^ | 0.152^b^ | - | - |
|  |  | 211G/A | 18.35 ± 9.10 | 17.21 ± 8.42 | 22.92 ± 11.26 | 26.80 ± 5.37 | 0.055^a^ | 0.046^b^ | 0.116^b^ | 0.649^b^ |
|  |  | 211A/A | 22.07 ± 11.21 | 22.59 ± 12.81 | 20.4 ± 2.87 | - | 0.714^b^ | 0.714^b^ |  |  |
|  |  | Compound  heterozygous  variation | 22.24 ± 6.40 | 22.93 ± 7.18 | 20.76 ± 4.37 | - | 0.472^b^ | 0.472^b^ | - | - |
| Incidence of  hyperbilirubinemia, (%) | *UGT1A1* | Wild-type | 28/109  (25.7%) | 24/96  (25%) | 1/10  (10%) | 3/3  (100%) | 0.007^c^ | 0.228^c^ | 0.004^c^ | 0.003^c^ |
|  |  | (TA)_6_/ (TA)_7_ | 32/63  (51.8%) | 23/47  (48.9%) | 9/15  (60%) | 0/1  (0%) | 0.448^c^ | - | - | - |
|  |  | (TA)_7_/(TA)_7_ | 12/12  (100%) | 9/9  (100%) | 3/3  (100%) | - | - | - | - | - |
|  |  | 211G/A | 40/77  (51.9%) | 30/63  (47.9%) | 8/12  (66.7%) | 2/2  (100%) | 0.186^c^ | - | - | - |
|  |  | 211A/A | 17/21  (81%) | 12/16  (75%) | 5/5  (100%) | - | 0.214^c^ | 0.214^c^ | - | - |
|  |  | Compound  heterozygous  variation | 17/22  (77.3%) | 12/15  (80%) | 5/7  (71.4%) | - | 0.655^c^ | 0.655^c^ | - | - |

^a-c^*P* value from ANOVA (a), student t test (b) and χ^2^ test (c).

Abbreviations: TB, total bilirubin.

**Figure S1. Total bilirubin values** (**log10-transformed) among genotypes of *UGT1A1* or *SLCO1B* gene**

**

**

Note：*: *P*<0.05；**: *P*<0.01.

TB, total bilirubin.
